# Challenges in the Treatment of Urinary Tract Infections: Antibiotic Resistance Profiles of *Escherichia coli* Strains Isolated from Young and Elderly Patients in a Southeastern Romanian Hospital

**DOI:** 10.3390/biomedicines13051066

**Published:** 2025-04-28

**Authors:** Andreea-Elena Topa, Constantin Ionescu, Anca Pinzaru, Elena Mocanu, Ana Maria Iancu, Elena Dumea, Bogdan Florentin Nitu, Florin Gabriel Panculescu, Simona Claudia Cambrea

**Affiliations:** 1 Clinical Hospital of Infectious Diseases, 900178 Constanta, Romania; denisanamaria@yahoo.com (A.M.I.); elenadumea@yahoo.com (E.D.); nitubogdan2008@gmail.com (B.F.N.); cambrea.claudia@gmail.com (S.C.C.); 2 Faculty of Medicine, Ovidius University from Constanta, 900470 Constanta, Romania; costin@anatomie.ro (C.I.); elena.mocanu@univ-ovidius.ro (E.M.); gabriel.panculescu@yahoo.ro (F.G.P.); 3 “St. Apostle Andrew” Emergency County Clinical Hospital, 900591 Constanta, Romania

**Keywords:** *Escherichia coli*, urinary tract infections, antimicrobial resistance, antibiotic stewardship

## Abstract

**Background/Objectives**: Urinary tract infections (UTIs) represent a significant public health challenge, with *Escherichia coli* being the primary causative pathogen. The rise in antimicrobial resistance (AMR), further intensified by shifts in antibiotic prescribing practices during and after the COVID-19 pandemic, poses substantial difficulties in treatment optimization and clinical management. **Methods**: This retrospective study analyzed 644 *E. coli* strains from urine samples collected in a southeastern Romanian hospital during two periods: pre-pandemic (2018–2019, N = 361) and post-pandemic (2023–2024, N = 283). Antimicrobial susceptibility was assessed using the VITEK automated system for key antibiotic classes. **Results**: A significant increase in fluoroquinolone resistance was observed, especially for ciprofloxacin (*p* = 0.02), alongside rising ceftriaxone resistance (*p* = 0.004), suggesting the spread of ESBL-producing strains. Resistance to trimethoprim/sulfamethoxazole, ampicillin, and amoxicillin/clavulanic acid remained high, limiting their empirical use. Carbapenem resistance was low (*p* > 0.1), while nitrofurantoin and fosfomycin retained high efficacy (*p* = 0.26 and *p* = 0.64). **Conclusions**: The post-pandemic period showed a concerning rise in resistance to fluoroquinolones and third-generation cephalosporins, highlighting the need for stricter antimicrobial stewardship. Carbapenems remain effective for severe infections, while nitrofurantoin and fosfomycin are reliable first-line options for uncomplicated UTIs. Continuous AMR surveillance is essential to optimize treatment and curb multidrug-resistant strains.

## 1. Introduction

Resistance to antibiotics has escalated significantly in recent years, now posing a genuine threat. Microorganisms like *Escherichia coli* presented a growing resistance even with last-generation antibiotics. Resistance is chiefly described as synthesizing beta-lactamase genes on mobile genetic elements, enabling their transference among various species. In rare instances, Gram-negative rods exhibit resistance to nearly all known antibiotics. The causes are manifold, but the overuse of antibiotics in humans is critical, along with the transmission of harmful germs in hospitals and communities, particularly through the food chain and contaminated hands. Moreover, novel antibiotics are scarce in development, especially for Gram-negative bacilli. The condition has improved marginally for Gram-positive cocci due to the introduction of several effective and innovative antibiotics in recent years. An essential need exists for a robust and coordinated multinational effort.

Antibiotic discovery has saved millions of lives and caused considerable changes in the medical industry. However, we are currently in a critical moment where antibiotics may no longer adequately treat bacterial illnesses [1,2]. Microorganisms are increasingly developing resistance to existing antibiotics, particularly Gram-negative rods such as *Escherichia coli*, which exhibit resistance to nearly all currently available antibiotics in certain contexts. This resistance may coalesce with virulence, forming a potentially lethal combination. Secondly, the antibiotic pipeline has become exceedingly barren [3]. In recent years, numerous potent compounds effective against Gram-positive cocci have been introduced; however, the same cannot be said for Gram-negative bacteria, and it is unlikely that any new antibiotic class targeting multi-resistant Gram-negative rods will emerge in the foreseeable future. Despite being challenging to conceive, the truth is that numerous practitioners may imminently encounter a therapeutic impasse in managing specific severe bacterial illnesses. Immediate action is not just necessary; it is a matter of life and death; inaction is not a solution [1,2].

Urinary tract infection (UTI) is a prevalent concern in pediatric and adults’ healthcare practitioners’ practical activity. In recent decades, the importance of urinary tract infections (UTIs) has been acknowledged, especially concerning their function as an overlooked cause of febrile illness in different age groups. The contemporary comprehension of urinary tract infections raises many inquiries and stimulates continuous discussion.

The current moment presents a dilemma: What will happen if antibiotic resistance increases in children and adults? This article aims to learn as much as possible about antibiotic resistance in urinary infections, regardless of age group.

Urinary tract infection (UTI) is the most dangerous bacterial illness in infancy, and numerous children with UTIs are hospitalized. Many of these children are administered antibiotics without an awareness of the causing pathogen or its susceptibility to the medication [4,5]. The resistance of uropathogens to antibiotics is escalating. Research in both adults and children has found several risk factors for resistant microbes, including previous antibiotic exposure and the administration of prophylactic antibiotics [4,5].

This study provides a valuable contribution by conducting a comparative analysis of the antimicrobial resistance profile of *E. coli* strains across two distinct periods—pre-pandemic and post-pandemic—highlighting the impact of the COVID-19 pandemic on antibiotic use and the emergence of resistant strains. Unlike previous studies that focus on general antimicrobial resistance trends, this research offers a detailed regional perspective on AMR dynamics within a hospital in southeastern Romania, with practical implications for optimizing UTI treatment and guiding antibiotic stewardship policies.

Antimicrobial resistance in *Escherichia coli* is driven by multiple mechanisms, including the production of extended-spectrum beta-lactamases (ESBL), chromosomal mutations, and plasmid-mediated resistance genes. These mechanisms contribute to the global spread of multidrug-resistant strains, which the World Health Organization has identified as a major public health priority.

## 2. Materials and Methods

A total of 644 *Escherichia coli* strains isolated from urine samples were included in this study, collected from patients hospitalized at the Clinical Hospital for Infectious Diseases Constanța (SCBI) during two distinct periods: pre-pandemic (2018–2019, N = 361) and post-pandemic (2023–2024, N = 283). For microbial isolation and cultivation, urine samples were inoculated on Blood Agar and Drigalski Lactose Agar and incubated at 37 °C for 16–24 h [6,7]. Bacterial identification was performed using the MALDI-TOF technique. Antibiotic susceptibility testing was conducted using the automated VITEK system, based on the microdilution method [6,7]. Phenotypic detection of ESBL- and carbapenemase-producing strains was also performed using the VITEK system. Results were interpreted according to the EUCAST guidelines available at the time of testing: EUCAST version 8.1 (2018) was used for isolates collected during the 2018–2019 period, and EUCAST version 13.0 (2023) was applied to isolates from the 2023–2024 period. Quality control was performed using the standard strain *Escherichia coli* ATCC 25922. The tested antimicrobial agents included ampicillin, amoxicillin/clavulanic acid, ceftriaxone, ertapenem, meropenem, levofloxacin, ciprofloxacin, nitrofurantoin, fosfomycin, and trimethoprim/sulfamethoxazole (TMP/SXM).

### 2.1. Inclusion and Exclusion Criteria

This retrospective study included patients aged between 1 and ≥65 years, diagnosed with microbiologically confirmed urinary tract infection, with a positive urine culture for *Escherichia coli* (≥10^5^ CFU/mL) and an available antibiotic susceptibility test. The study period was divided into two intervals: pre-pandemic (2018–2019) and post-pandemic (2023–2024).

**Inclusion criteria:** Age between 1 and ≥65 years, clinical and microbiological diagnosis of urinary tract infection, *E. coli* identified as the primary etiologic agent, availability of data on prior antibiotic treatments, patients diagnosed with HIV with a CD4 cell count above 500 cells/mm^3^.

**Exclusion Criteria**: Mixed urinary tract infections (presence of other significant pathogens), nosocomial infections, major renal-urinary anomalies (e.g., vesicoureteral reflux grade ≥ III), severe immunosuppression (e.g., transplant, neutropenia, neoplasia), unavailability of antibiogram results or incomplete clinical/microbiological data, as well as duplicate isolates from the same patient during the same infectious episode.

The origin of infection (community-acquired vs. hospital-acquired) was not included as an eligibility criterion due to informational limitations of the dataset and was therefore not used in the comparative analysis.

### 2.2. Statistical Analysis

For the statistical description of the data, absolute and relative frequencies were calculated for categorical variables, while the median and interquartile range (IQR) were used for numerical variables. Group comparisons (univariate analysis) were conducted using the Chi-square (χ^2^) test for categorical variables, including resistance rates. Fisher’s exact test was applied only when the assumptions for the Chi-square test were not met. For continuous variables, such as age, the independent samples *t*-test was applied when appropriate. The significance threshold was set at *p* < 0.05. Primary data collection was performed using Microsoft Excel (Microsoft^®^ Excel^®^ for Microsoft 365 MSO, version 2501), while statistical analysis was conducted using JAMOVI, version 2.6.24.0.

## 3. Results

### 3.1. Patient Characteristics

To underscore the rise in antibiotic resistance, we conducted the study before and after the epidemic’s isolation period. Consequently, two distinct timeframes are emphasized: 2018–2019, representing the pre-pandemic era, and 2023–2024, denoting the post-pandemic phase. The intricacy of the examined cohort (children and adults) necessitated the rigorous enforcement of the inclusion criteria. Consequently, after removing all individuals with chronic or autoimmune disorders, structural anomalies, and those unwilling to sign the informed consent, we delineated a cohort of 644 patients. Table 1 describes the comparison of the etiological profiles between males and females across various age categories. The etiological patterns across several age categories exhibited divergence, categorized by gender (male and female) and ward type (emergency). The age groups are divided: children 1–18 years old, adults 19–64 years old, and geriatrics over 65 years old (Table 1). The age distribution of isolates was as follows: Children (8.69%), 19–64 (57.91%), and ≥65 (33.38%) years.

We highlight that 90.2% of the participants originate from an urban setting. Romania is a nation in ongoing development, but it still has not improved medical care in rural regions. As a result, access to medical care in these areas continues to pose a barrier. The group comprised 207 persons with one UTI, 236 with two to three UTIs, and 201 with four or more UTIs during the study period. The female proportion and mean age increased with each consecutive UTI. Individuals with a single uncomplicated urinary tract infection (UTI) were predominantly adults without underlying comorbidities (45%), followed by the geriatric population (34%) and children (8.69%).

The distribution of patients (Table 2) shows a high prevalence of cases in the adult infectious diseases ward (69.25%), followed by immunocompromised patients (16.77%), pediatrics (6.67%), and the intensive care unit (ICU) (2.17%). These findings suggest a significant impact of *E. coli* infections on the adult and immunocompromised populations, emphasizing the need for tailored prevention and treatment strategies, as well as continuous antimicrobial resistance surveillance in these vulnerable groups.

### 3.2. Resistance Profile of E. coli Strains

The results indicate a modest increase in the prevalence of extended-spectrum β-lactamase (ESBL)-producing strains, from 10.8% before the pandemic to 13.42% in the post-pandemic period, without reaching statistical significance (*p* = 0.3) (Table 3). Regarding carbapenemase-producing strains, prevalence remained extremely low, with only two cases identified across the entire study cohort (0.31%), showing no significant differences between the two time periods (*p* = 0.8). These findings suggest that, although the COVID-19 pandemic was associated with significant changes in antibiotic use and selective pressure on pathogenic microorganisms, *E. coli* resistance to carbapenems remained limited, while the increase in ESBL production was modest and not statistically significant. The data emphasize the need for ongoing antimicrobial resistance surveillance strategies and prudent antibiotic use policies.

Although the comparative assessment between the pre-pandemic (2018–2019) and post-pandemic (2023–2024) periods did not reveal statistically significant differences for all analyzed antibiotics, emerging trends indicate a subtle progression of antimicrobial resistance (Table 4). The increasing proportion of MDR strains (*p* = 0.026) constitutes a critical warning signal that necessitates immediate and stringent measures to regulate antibiotic use.

Resistance to ampicillin and amoxicillin/clavulanic acid remained low in pediatric patients (5.3% and 6.7%, respectively) but reached concerning levels in adults (38.5% and 30.4%) and elderly patients (18.3% and 20.5%). Notably, elderly patients and males exhibited a sharp increase in resistance to third-generation cephalosporins, suggesting a possible selection pressure driven by excessive antibiotic use in these vulnerable populations.

This investigation highlighted statistically significant variations in age-specific susceptibilities of *E. coli* to ampicillin and amoxicillin-clavulanate between males and females. Urinary *E. coli* isolates from female patients demonstrated higher antibiotic resistance than male patients. Meanwhile, the time trends and age-specific susceptibility differences were statistically significant. However, these discrepancies must be viewed cautiously, as this investigation only assessed susceptibility to amoxicillin-clavulanate for ampicillin non-susceptible *E. coli* isolates.

A significant rise in fluoroquinolone resistance (levofloxacin, ciprofloxacin) was observed post-pandemic, particularly among hospitalized patients, possibly reflecting excessive and inappropriate antibiotic use during the pandemic. This phenomenon sets the stage for a dangerous scenario where standard treatments for complicated UTIs may become ineffective.

Women remain the predominant group affected by UTIs (>80% of cases), but an alarming increase in cephalosporin resistance has been noted in men. These gender-related differences in AMR profiles suggest that both biological factors and antibiotic prescribing patterns contribute to disparities in the selection of resistant strains.

Although an immediate explosion of resistance post-pandemic was not observed, the data indicate a subtle shift in AMR dynamics, with a gradual accumulation of MDR strains. This may be a consequence of uncontrolled antibiotic use during the pandemic, underscoring the urgent need for strict antimicrobial stewardship policies.

## 4. Discussion

The global proliferation of antibiotic resistance among uropathogens responsible for urinary tract infections is concerning. This resistance can transform ostensibly little ailments into perilous infections, potentially resulting in serious health problems. *E. coli* is a good indicator bacterium for testing antibiotic resistance since it is widespread in urinary tract infections and susceptible to conventional treatments. Antibiotic resistance prevention is vital for healthcare professionals, medical students, and health enthusiasts. This study presents the importance of urinary tract infections and the antibiotic susceptibility of uropathogens in Constanta, Romania before and after the pandemic period. Consistent with prior research conducted in Europe, *E. coli* continues to be the predominant pathogen isolated from urine cultures in pediatric, adult, and geriatric populations [8,9,10,11,12,13,14,15,16,17,18].

**ESBL- and Carbapenemase-Producing *E. coli* Strains**

This study analyzed the evolution of *E. coli* antibiotic resistance for carbapenem over two distinct periods, pre-pandemic (2018–2019) and post-pandemic (2023–2024), in a total sample of 644 patients. The results indicate a modest increase in the prevalence of extended-spectrum β-lactamase (ESBL)-producing strains, from 10.8% before the pandemic to 13.42% in the post-pandemic period, without reaching statistical significance (*p* = 0.3). Altamimi et al. [19] emphasized the same data obtained in this study related to the prevalence of and resistance to carbapenem. The data obtained are the same regardless of the analyzed period.

Prior research, including that of Bradford et al. and Mena et al. [20,21], has indicated an increasing prevalence of ESBL-producing bacteria, especially in *E. coli*. Regarding carbapenem-producing strains, prevalence remained extremely low, with only two cases identified across the entire study cohort (0.31%), showing no significant differences between the two time periods (*p* = 0.8). Wardoyo et al. [22] demonstrated a decrease in ESBL production in *E. coli*. This presents strong evidence that the COVID-19 pandemic may have notably influenced the dynamics of antimicrobial resistance among different demographic groups and healthcare environments in specific regions.

This particular case deviates from global trends of rising antibiotic resistance. These findings suggest that, although the COVID-19 pandemic was associated with significant changes in antibiotic use and selective pressure on pathogenic microorganisms, *E. coli* resistance to carbapenems remained limited while the increase in ESBL production was modest and not statistically significant. The data emphasize the need for ongoing antimicrobial resistance surveillance strategies and prudent antibiotic use policies.

**Resistance of *E. coli* Strains to the Aminopenicillin Group**

The analysis of the antimicrobial resistance profile of *Escherichia coli* strains from the pre-pandemic (2018–2019) and post-pandemic (2023–2024) periods indicates minor variations, with no statistically significant changes observed for most antibiotics examined. Resistance to ampicillin was low in children (5.90%) compared to adults (34.93%) and the geriatric group (19.25%), with no significant differences observed between the two study periods (*p* = 0.25). A comparable trend was noted for amoxicillin/clavulanic acid, with resistance rates being lower in children (5.59%) compared to adults (31.36%) and elderly patients (17.23%), and no significant increasing trend was observed in the post-pandemic period (*p* = 0.11). Resistance to ampicillin is noted in Romania as well but with different results as in this study by Golli et al. [23] *Escherichia coli* emerged as the most commonly isolated bacterium, exhibiting the highest resistance to amoxicillin/clavulanic acid and second-generation cephalosporins. Before the pandemic, in Poland, this microorganism’s resistance to aminopenicillins among adults and the geriatric population was higher than the European average. Our results indicate that the percentage of ampicillin-resistant strains was 34%, aligning closely with the European average [24,25] Rodríguez-Lozano et al. demonstrate that resistance to ampicillin, amoxicillin-clavulanate, and co-trimoxazole was significant, exceeding 20%, in isolates from pediatric patients older than 1 month and adults [26]. The data obtained for ampicillin and cotrimoxazole are consistent with the international literature, supporting the data obtained in the study [27,28,29,30].

**Resistance Profile of *E. coli* Strains to Third-Generation Cephalosporins**

The resistance to ceftriaxone exhibited a notable increase post-pandemic, rising from 5.5% to 8.1% among adults and from 2.8% to 8.5% among geriatric patients (*p* = 0.004). A slight decrease in the proportion of resistant strains was observed in children, declining from 0.8% to 0.35%. The findings indicate a potential impact of alterations in therapeutic practices and antibiotic utilization during and subsequent to the COVID-19 pandemic. Before the pandemic period this study identified moderate resistance to cefixime and other third-generation cephalosporins, consistent with findings from another researcher [31,32,33]. Conversely, the resistance to cefotaxime and other third-generation cephalosporins in Austria was minimal [34]. Mehr et al. [35] highlighted the need for immediate action to address antibiotic resistance among children under six years of age before the pandemic. They reported that resistance to ampicillin/amoxicillin was observed in 52% of isolates, trimethoprim in 14%, and cephalothin/cephalexin in 24%. The highest recorded resistance rate to first-generation cephalosporins was found in Australia’s adult and pediatric urinary tract infections. Shimoni et al. recommended empiric antibiotic therapy with a second- or third-generation cephalosporin for elderly patients with a urinary tract infection (UTI), resulting in a reduction in hospitalization days and potentially mortality [36].

Preliminary data reveal that overusing self-administered over-the-counter antibiotics and physician-prescribed medications for COVID-19 inpatients has altered bacteria resistance patterns. Knowing the resistance trend of the most frequent uropathogens is crucial for the optimum treatment of these illnesses [37].

**Susceptibility of *E. coli* Strains to the Carbapenem Class**

Resistance to ertapenem and meropenem among carbapenems has remained notably low, with no significant increase post-pandemic (*p* = 0.14 and *p* = 0.63, respectively). Significantly, no resistant strains were identified in adolescents, which is a reassuring indication for the health of future generations. Minor changes were observed in adults and elderly individuals, but the overall results indicate the continued effectiveness of carbapenems and a restricted dissemination of carbapenemase-producing bacteria.

The prevalence of carbapenemase-producing isolates is minimal in our region. A single isolate was identified in the urine culture of a young patient, exhibiting no significant changes compared to those from older patients.

In the present investigation, carbapenem resistance exhibits a linear trend over the two periods. This is in contrast to findings from other investigations, including those by Nordmann et al. [38] and others [19,37,39,40], which indicated a worldwide increase in carbapenem resistance. Unlike earlier research, a Romanian study emphasized a consistent sensitivity to carbapenems in *E. coli* during the observation period from 2018 to 2022 [41]. The explanation of this linear trend is not reassuring because it suggests the pandemic period. However, it limited access to antibiotics and did not reduce unnecessary exposure to their consumption. This distinction underscores the dynamic characteristics of antibiotic resistance.

**Resistance Profile of *E. coli* Strains to the Fluoroquinolone Group**

A comparative analysis of *Escherichia coli* resistance to fluoroquinolones between the pre-pandemic (2018–2019) and post-pandemic (2023–2024) periods reveals a significant increase in ciprofloxacin resistance (*p* = 0.02) and an upward trend for levofloxacin resistance (*p* = 0.36), particularly among adults and elderly patients. These findings suggest a possible impact of changes in therapeutic practices during the pandemic, with increased use of these antibiotics favoring the selection of resistant strains. Among children, resistance to both fluoroquinolones remained relatively stable, reflecting the more restricted use of this antibiotic class in pediatric practice.

A recent systematic review of *E. coli* fluoroquinolone resistance in UTIs in women found that ciprofloxacin resistance in isolates increases over time, especially in the UK, Spain, and Germany [42]. In Asia, ciprofloxacin resistance increased by 40% in 2014, with Bangladesh having the highest rate (69%) [43]. Even more, two studies reported considerable fluoroquinolone resistance in Iran (55.6%) [42,44] and Romania (15%) [42,45]. A 2012–2019 Chinese retrospective analysis found ciprofloxacin resistance in UPEC ranging from 55% to 70% [46]. North American resistance rose to 12% in 2017 [42,47]. Our research found a significant increase, but higher rates were noted in Canada [48]. The FDA warning on fluoroquinolone use may have reduced fluoroquinolone resistance, which is speculative but of interest for future investigations in this country [42]. Turkish individuals with UTIs had fluoroquinolone resistance rates of 52% in 2020, up from 20–30% in 1996 [49]. A Mexican investigation found that less than 40% of identified isolates were ciprofloxacin-resistant, except for ESBL producers [42,50]. Venugopal et al. [51] published increased minimum inhibitory concentration (MIC50) to beta-lactams and fluoroquinolones seen during the pandemic, which contrasts with the pre-pandemic period.

The worldwide proliferation of multidrug-resistant germs has resulted in a rise in challenging-to-treat urinary tract infections, a trend also noted in pediatric populations [52,53]. The increase in multi-resistant uropathogens complicates the management of these illnesses [52,53]. MDR infections can be either community-acquired or hospital-acquired. Our findings diverge marginally from those of Miftode et al., who reported MDR rates of 60.3%, for *E. coli* [53]. The utilization of fluoroquinolones in pediatric populations remains minimal; nonetheless, certain institutions are documenting a rise in their application [52,54,55,56]. The data indicate that bacterial isolates from the urinary tracts of children with previous antibiotic prescriptions had a higher likelihood of antibiotic resistance [52].

**Resistance to Nitrofurantoin and Fosfomycin**

An analysis of 644 patients tested for *Escherichia coli* susceptibility to nitrofurantoin and fosfomycin during the pre-pandemic (2018–2019, N = 361) and post-pandemic (2023–2024, N = 283) periods shows consistently low resistance rates, with no statistically significant differences (*p* = 0.26 for nitrofurantoin, *p* = 0.64 for fosfomycin). Nitrofurantoin resistance remained low, at 1.4% pre-pandemic and 2.8% post-pandemic, while fosfomycin resistance was 6.6% pre-pandemic and 7.8% post-pandemic. These findings confirm that nitrofurantoin and fosfomycin continue to be effective therapeutic options for uncomplicated urinary tract infections, particularly in children and adults.

Nitrofurantoin was the initial extraordinarily successful and safe antibiotic administered for urinary tract infections; however, numerous bacterial pathogens have developed resistance to it throughout the decades [57]. The incidence of nitrofurantoin resistance fluctuated over the years, as reported in various research compiled by Akter et al.: starting from 10% (1987) and going up to 73.7% (2013) [57,58,59]. Nevertheless, it is increasingly utilized following its cessation, resulting in a microbial resistance. Numerous investigations have documented limited bacterial resistance (0–5%) globally despite the prolonged use of nitrofurantoin [57,58,59,60]. Nitrofurantoin appears to be especially effective for treating urinary tract infections due to its high concentration in urine [61].

Fosfomycin is sporadically utilized in pediatric medicine. Gul et al. [15] assessed urinary infections and found that *E. coli* exhibited decreased resistance rates to ampicillin, fosfomycin, and nitrofurantoin. Post-pandemic international literature discusses children with urinary tract malformations, particularly those with a history of urological interventions, regarding recurrent UTIs caused by antibiotic-resistant strains; several months of non-standard prophylactic treatment with fosfomycin may be contemplated [62].

**Resistance of *E. coli* Strains to Trimethoprim/Sulfamethoxazole (TMP/SMX)**

The analysis of *Escherichia coli* resistance to trimethoprim/sulfamethoxazole (TMP/SMX) over the two study periods shows consistently high resistance rates, with no statistically significant differences. In the pre-pandemic period (2018–2019), resistance was 39%, while in the post-pandemic period (2023–2024), it was 37.1% (*p* = 0.62). These findings confirm the limited efficacy of TMP/SMX as an empirical treatment option for UTIs and emphasize the importance of antimicrobial susceptibility testing before its use.

Recent data on antibiotic resistance trends across European nations indicate concerning outcomes regarding *E. coli* resistance rates to aminopenicillins, fluoroquinolones, and trimethoprim/sulfamethoxazole; however, favorable sensitivity profiles were noted for aminoglycosides, carbapenems, and cephalosporins [37].

Recent NICE guidelines now advocate for the use of nitrofurantoin over trimethoprim as the first-line treatment for uncomplicated urinary tract infections in adults; however, a similar recommendation has not yet been established for children, likely due to the limited resistance data available specifically for this demographic in the UK. Bryce et al. [63] presented the cumulative prevalence of *E. coli* resistance to 53.4% for ampicillin, 23.6% for trimethoprim, 8.2% for co-amoxiclav, and 2.1% for ciprofloxacin, with nitrofurantoin exhibiting the lowest resistance at 1.3%. These findings underscore the urgent need for further research and the development of new clinical practices [52].

This study’s findings indicate significant resistance to frequently administered antibiotics in pediatric primary care [15]. The overall incidence of resistance was most significant against trimethoprim, all of which are advised as first-line therapies for urinary tract infections in children. Consistent with the guidelines from infectious disease organizations in the USA and Europe advocating a 20% resistance threshold for first-line UTI treatment [15,62] certain antibiotics, especially amoxicillin, should no longer be endorsed as first-line therapy for UTI in the UK. However, it is garnering heightened interest due to escalating rates of antibiotic resistance. It is crucial to ascertain whether isolates acquire resistance during infection under antimicrobial pressure or prior to infection while acting as colonizers [15,62].

This study demonstrates a significant increase in *E. coli* resistance to fluoroquinolones and third-generation cephalosporins in the post-pandemic period, with major clinical implications for the management of urinary tract infections (UTIs). This trend highlights the impact of empirical antibiotic use and the urgent need for stricter antimicrobial stewardship policies [30,64,65,66].

This increase may be attributed to several contributing factors. The COVID-19 pandemic led to a rise in empirical antibiotic use, particularly broad-spectrum agents, in both hospital and outpatient settings. In many cases, antibiotics were prescribed or self-administered in the absence of confirmed bacterial infections, driven by uncertainty and limited diagnostic access. Moreover, the strain placed on healthcare systems during the pandemic likely compromised infection control protocols, facilitating the spread of resistant strains. These combined factors may have exerted selective pressure on uropathogenic *E. coli*, contributing to the resistance trends observed in this study.

Analyzing data from the specialized literature, we can definitely say that there is data that support the correlation between resistance trends to ceftriaxone and fluoroquinolones and the presence of *E. coli* strains producing extended-spectrum beta-lactamases, especially those with a specific genetic background. Ceftriaxone resistance is strongly associated with the presence of ESBL genes, in particular: -blaCTX-M (subtypes CTX-M-15, CTX-M-14, CTX-M-27). These genes are frequently found on conjugated plasmids, which favors horizontal transmission between strains and species. Horizontal transmission refers to the transfer of genetic material between different strains or species, which can lead to the spread of antibiotic resistance. CTX-M-15, a subtype of blaCTX-M, is not a rare find in *E. coli* strains. In fact, it is a frequent companion, often associated with urinary and systemic infections. This prevalence underscores the urgent need to understand and address this issue. Resistance to fluoroquinolones is often mediated by: chromosomal mutations in the gyrA and parC genes, which affect topoisomerase II and IV; plasmid resistance genes such as qnr or aac. In particular, *E. coli* clones ST 131 H30-Rx are recognized for their resistance. Molecular studies show that the prevalence of ESBL *E. coli* with the genetic background ST 131 and its variants has increased over the past two decades in many regions of the world. Genetic WGS or multiplex PCR monitoring confirms these associations in different cohorts and surveillance systems [67,68].

Age-group analysis revealed notable differences in resistance profiles. The highest increase in fluoroquinolone and ceftriaxone resistance was observed in adults and elderly patients during the post-pandemic period, suggesting a possible correlation with increased antibiotic exposure. In contrast, resistance in children remained relatively stable, likely due to more restrictive antibiotic use in pediatrics. Resistance to ampicillin and trimethoprim/sulfamethoxazole (TMP/SMX) remained high across all age groups, limiting their use as empirical treatment options.

Carbapenems have maintained their efficacy, remaining a crucial last-resort option for severe infections; however, their use must be closely monitored to prevent the emergence of carbapenemase-producing strains [66]. Additionally, nitrofurantoin and fosfomycin continue to be effective antibiotics for uncomplicated UTIs, with consistently low resistance rates, supporting their recommendation as first-line therapy according to international guidelines [30].

A study conducted by Cambrea (2015) and published in the Journal of Pediatric Infectious Diseases investigated the antibiotic resistance profile of *E. coli* strains isolated from pediatric patients in our clinic [69]. The findings revealed a high prevalence of resistance to ampicillin, followed by trimethoprim/sulfamethoxazole (SXT). In comparison to the current study, an analysis of the two time periods demonstrated an increasing trend in resistance among *E. coli* isolates from urine cultures to ampicillin and SXT.

The range of microorganisms responsible for severe diseases is broadened in the southeastern region of Romania. Cambrea et al., in conjunction with Halichidis et al. [70,71,72], elucidated through intricate analyses the significance of environmental factors on the organism and the heightened risk of sepsis, even when antibiotics are promptly administered. However, the rise in resistance has rendered these treatments ineffective, underscoring the urgent need for alternative solutions.

The intricacy of the human body and the potential for intrainfectious events are significantly influenced by renal abnormalities. Notably, a vascular structural abnormality affecting the parenchyma was observed in two adult participants with recurrent urine infections (three episodes per individual). This is particularly relevant to our audience of medical professionals and researchers interested in renal health and infectious diseases. Matusz et al. have also addressed this subject in the local literature, and therefore it is not seen as an exception [73].

## 5. Study Limitations

These findings contribute to a better understanding of post-pandemic changes in the antimicrobial resistance profiles of *Escherichia coli* strains isolated from urinary tract infections. However, the study presents several important limitations that should be considered when interpreting the results. Clinical data such as comorbidities or prior antibiotic use were not available, and a clear distinction between community-acquired and hospital-acquired infections could not be made, as this variable was not used as a selection criterion. Additionally, although molecular/genetic testing could not be performed due to financial constraints and a lack of equipment, phenotypic confirmation of ESBL- and carbapenemase-producing strains was carried out using the VITEK automated system, providing valuable information on the resistance mechanisms involved. Therefore, our understanding of the relationship between bacterial genetic structure and resistance patterns is based on data extrapolated from the international literature. These limitations, particularly the absence of molecular/genotypic characterization, play a significant role in shaping the scope and interpretation of this research.

## 6. Conclusions

These findings emphasize the importance of implementing effective antibiotic stewardship strategies and rapid bacterial susceptibility testing to limit the spread of multidrug-resistant strains and optimize antimicrobial treatments. The continuous surveillance of antimicrobial resistance is essential for guiding therapeutic decisions and preventing the escalation of antibiotic resistance [30,64].

## Figures and Tables

**Table 1:** Distribution of *E. coli* Strains by Demographic Characteristics.

		Overall N = 644	Pre-Pandemic 2018–2019 N = 361	Post-Pandemic 2023–2024 N = 283	*p*-Value
**Category of age**	**Sex, n (%)**				
**Adults** **(19–64 ani)**	Female	293 (45.49)	182 (50.41)	111 (39.22)	0.031
	Male	80 (12.42)	39 (10.80)	41 (14.48)	
**Children** **(1–18 ani)**	Female	46 (7.14)	22 (6.09)	24 (8.48)	0.9
	Male	10 (1.55)	5 (1.38)	5 (1.76)	
**Older adult** **(≥65 ani)**	Female	188 (29.19)	95 (26.31)	93 (32.87)	0.116
	Male	27 (4.19)	18 (4.99)	9 (3.18)	
	**Age, Median (IQR)**	53	51	55	0.08

**Table 2:** Distribution of *E. coli* Strains by Hospital Ward.

	Overall N = 644	Pre-Pandemic 2018–2019 N = 361	Post-Pandemic 2023–2024 N = 283	*p*-Value
**Ward, n (%)**				<0.001
**Adults ID**	446 (69.25)	291 (80.61)	155 (54.7)	
**Childrent ID**	43 (6.67)	19 (5.26)	24 (8.48)	
**ICU-ID**	14 (2.17)	5 (1.38)	9 (3.18)	
**Emergency**	25 (3.88)	2 (0.55)	23 (8.13)	
**Imunodepressed** **(Human Immunodeficiency virus)**	108 (16.77)	43 (11.91)	65 (22.9)	
**Others**	8 (1.24)	1 (0.27)	7 (2.47)	

**Table 3:** ESBL- and Carbapenemase-Producing *E. coli* Strains.

	Overall N = 644	Pre-Pandemic 2018–2019 N = 361	Post-Pandemic 2023–2024 N = 283	*p*-Value
**ESBL, n (%)**				0.3
**Negative**	567 (88.04)	322 (89.19)	245 (86.57)	
**Positive**	77 (11.95)	39 (10.80)	38 (13.42)	
**Carbapenemase, n (%)**				0.8
**Negative**	642 (99.7)	360 (99.72)	282 (99.64)	
**Positive**	2 (0.31)	1 (0.27)	1 (0.35)	

**Table 4:** Patients’ demographic characteristics for 644 *E. coli* urinary tract isolates during the pre-pandemic (2018–2019, N = 361) and post-pandemic (2023–2024, N = 283) periods.

Pre-Pandemic 2018–2019														
	Total No Isolates/Susceptibility to All Antibiotic/Resistance to One or More N (%) Isolate Resistance to													
				AMP	AMC	CRO	ERT	MEM	LVX	CIP	NIT	FOS	SXT	%MDR
**Gender**														
**Male**	62 (17.2)	16 (4.4)	46 (12.74)	40 (11.1)	38 (10.5)	8 (2.2)	2 (0.6)	0 (NA)	19 (5.3)	19 (5.3)	3 (0.8)	4 (1.11)	24 (6.64)	8 (2.21)
**Female**	299 (82.8)	89 (24.6)	210 (58.17)	184 (50.9)	168 (46.5)	25 (6.9)	4 (0.4)	3 (0.3)	70 (19.4)	48 (13.3)	2 (0.6)	20 (5.5)	117 (32.4)	16 (4.4)
**Total**	361	105 (29.1)	256 (19.6)	224 (61.5)	206 (57.1)	33 (9.1)	6 (1.7)	3 (0.8)	89 (24.6)	67 (18.5)	5 (1.4)	24 (6.6)	141 (39)	24 (6.6)
**Category of age**														
**Children** **1–18 years**	27 (7.5)	4 (1.1)	8 (2.2)	19 (5.3)	20 (5.54)	3 (0.8)	0 (NA)	0 (NA)	7 (1.9)	5 (1.4)	0 (NA)	3 (0.8)	12 (3.3)	2 (0.5)
**Adults** **19–64 years**	221 (61.2)	66 (18.3)	162 (44.9)	139 (38.5)	126 (34.6)	20 (5.5)	2 (0.5)	1 (0.2)	50 (14)	36 (10)	4 (1.1)	15 (4.1)	84 (23.3)	16 (4.4)
**Geriatrics** **≥65 years**	113 (31.3)	35 (9.6)	86 (23.9)	66 (18.3)	60 (16.6)	10 (2.8)	4 (1.1)	2 (0.6)	32 (8.8)	26 (7.2)	1 (0.2)	6 (1.7)	45 (12.4)	6 (1.7)
**Total**	361	105	256	224	206	33	6	3	89	67	5	24	141	24
**Post-pandemic 2023–2024**														
**Gender**														
**Male**	55 (19.4)	10 (3.4)	41 (14.4)	41 (14.5)	35 (12.4)	16 (5.6)	1 (0.35)	1 (0.35)	28 (9.9)	27 (9.5)	2 (0.7)	4 (1.4)	37 (13.1)	13 (4.6)
**Female**	228 (80.6)	79 (28)	153 (54.1)	122 (43.1)	108 (38.1)	32 (11.3)	0 (NA)	0 (NA)	51 (18)	47 (16.6)	6 (2.1)	18 (6.4)	68 (24)	21 (7.4)
**Total**	283	89 (31.4)	194 (68.5)	163 (57.6)	143 (50.5)	48 (16.9)	1 (0.35)	1 (0.35)	79 (27.9)	74 (26.1)	8 (2.8)	22 (7.8)	105 (37.1)	34 (12)
**Category of age**														
**Children** **1–18 years**	29 (10.2)	6 (2.1)	19 (6.7)	19 (6.7)	16 (5.6)	1 (0.35)	0 (NA)	0 (NA)	6 (2.1)	6 (2.1)	0 (NA)	1 (0.35)	13 (4.6)	1 (0.35)
**Adults** **19–64 years**	152 (53.7)	51 (18)	101 (35.7)	86 (30.4)	76 (26.8)	23 (8.1)	0 (NA)	0 (NA)	42 (14.9)	42 (14.9)	4 (1.4)	8 (2.8)	58 (20.5)	19 (6.7)
**Geriatrics** **≥65 years**	102 (36)	32 (11.3)	74 (26.1)	58 (20.5)	51 (18.1)	24 (8.5)	1 (0.35)	1 (0.35)	31 (10.9)	26 (9.2)	4 (1.4)	13 (4.6)	34 (12)	14 (4.95)
**Total**	283	89	194	163	143	48	1	1	79	74	8	22	105	34
***p*** **-value**				**0.25**	**0.11**	**0.004**	**0.14**	**0.63**	**0.36**	**0.02**	**0.26**	**0.64**	**0.62**	**0.026**

AMP—Ampicillin; AMC—Amoxicilin/clavulanic acid, CRO-Ceftriaxone, ERT—Ertapenem, MEM—Meropenem, LVX—Levofloxacina, CIP—Ciprofloxacin, NIT—Nitrofurantoin, FOS—Fosfomycin, SXT—Trimethoprim/sulfamethoxazole.

## Data Availability

The data supporting the reported results are available from the Ethics Commission, with approval number 12/25.03.2025. Due to ethical reasons, access to the data is restricted; however, they can be made available upon request through the Ethics Commission.

## Abbreviations

The following abbreviations are used in this manuscript:

**AMP**	Ampicillin
**AMC**	Amoxicilin/clavulanic acid
**CRO**	Ceftriaxone
**ERT**	Ertapenem
**MEM**	Meropenem
**LVX**	Levofloxacina
**CIP**	Ciprofloxacin
**NIT**	Nitrofurantoin,
**FOS**	Fosfomycin
**SXT** **TMP/SMX**	Trimethoprim/sulfamethoxazole
**MDR**	Multidrug resistance
